# The role of anticipated emotions in self-control: linking self-control and the anticipatory ability to engage emotions associated with upcoming events

**DOI:** 10.3389/fpsyg.2023.1152155

**Published:** 2024-01-08

**Authors:** Johann D. Kruschwitz, Thomas Goschke, Elkhansa Ahmed Mohamed Ali, Anne-Carolin Kraehe, Franziska Maria Korb, Henrik Walter

**Affiliations:** ^1^Division of Mind and Brain Research, Department of Psychiatry and Psychotherapy, Charité-Universitätsmedizin Berlin, Berlin, Germany; ^2^Collaborative Research Centre (SFB 940) "Volition and Cognitive Control", Technische Universität Dresden, Dresden, Germany; ^3^Department of Psychology, Technische Universität Dresden, Dresden, Germany

**Keywords:** self-control, anticipated emotions, dual system view, emotion regulation, volitional control

## Abstract

Self-control is typically attributed to “cold” cognitive control mechanisms that top-down influence “hot” affective impulses or emotions. In this study we tested an alternative view, assuming that self-control also rests on the ability to anticipate emotions directed toward future consequences. Using a behavioral within-subject design including an emotion regulation task measuring the ability to voluntarily engage anticipated emotions towards an upcoming event and a self-control task in which subjects were confronted with a variety of everyday conflict situations, we examined the relationship between self-control and anticipated emotions. We found that those individuals (*n* = 33 healthy individuals from the general population) who were better able to engage anticipated emotions to an upcoming event showed stronger levels of self-control in situations where it was necessary to resist short-term temptations or to endure short-term aversions to achieve long-term goals. This finding suggests that anticipated emotions may play a functional role in self-control-relevant deliberations with respect to possible future consequences and are not only inhibited top-down as implied by “dual system” views on self-control.

## 1 Introduction

In our everyday lives, we often experience situations that require self-control – that is, the ability to resist temptations or to endure aversive situations in order to achieve long-term goals (Baumeister et al., 2007; Hassin et al., 2010). For example, foregoing a delicious dessert or getting up to exercise after a hard day at work. Although self-control may seem like a routine skill, it is one of the most crucial prerequisites for personal autonomy (Locke, 1690) and one of the least understood functions of the human brain to date. The importance of this ability becomes particularly apparent when self-control fails and individuals act in conflict with their long-term goals and intentions; for example, in addictive behaviors characterized by a progressive loss of control over one’s own behavior despite awareness of the negative long-term consequences (Baler and Volkow, 2006). Conflicts between long-term goals and impulsive responses to immediate rewards can be understood as a byproduct of the rapid expansion of anticipatory abilities in the evolution of human cognition (Schoenemann et al., 2005; Teffer and Semendeferi, 2012; Hofman, 2014). Thus, the basic ability to associate actions with their consequences has evolved into a multifaceted range of anticipatory abilities, including episodic future thinking, mental time travel, or planning and anticipating actions that are distant in time and space (*cf.* Suddendorf and Corballis, 2007; Baumeister et al., 2011). These mental abilities are a crucial prerequisite for self-control, as they enable individuals to pursue goals that are motivated not by current but anticipated future needs (Kuhl and Goschke, 1994). Such long-term goals often conflict with impulsive reactions or current needs, e.g., when the intention to follow a diet is undermined by the sight of a tasty dessert. In such conflicts, self-control is required to resist temptations, inhibit impulsive reactions, and accept short-term costs (Hofmann et al., 2009a; Mischel et al., 2011).

Although impressive progress has been made in uncovering the neural systems underlying self-control (for reviews, see Inzlicht et al., 2014; Kelley et al., 2015; Han et al., 2018; Turner et al., 2019), many key questions about their specific mechanisms remain largely unresolved. A dominant hypothesis derives from “dual system theories”, which conceive of human decision-making as the result of a competition between an “impulsive” system, which responds selectively to immediate reward and strongly discounts future rewards, and a cognitive control system, which promotes anticipation of long-term goals and minimally discounts delayed rewards (e.g., Loewenstein, 1996; Metcalfe and Mischel, 1999; Strack and Deutsch, 2004; Hofmann et al., 2009b; Kahneman, 2011; Evans and Stanovich, 2013; McClure and Bickel, 2014; Volkow and Baler, 2015; Evans, 2019; Lindgren et al., 2019). One system is seen as governing the selection and control of actions by deliberative processes, long-term goals, and anticipated future outcomes. The second system mediates either impulsive or habitual responses, which are based on current desires or direct stimulus–response associations, respectively. Accordingly, at a neural level, dual systems theorists have attributed self-control to “cold” cognitive control mechanisms mediated by regions in the lateral prefrontal and parietal cortex, which are thought to either compete with “hot” affective impulses (e.g., McClure et al., 2004). Neuroimaging evidence for dual valuation systems stems from fMRI studies of intertemporal choice tasks, in which participants were asked to choose between smaller sooner and larger later rewards. Initial studies revealed that choosing immediate rewards was primarily associated with brain activation in mesolimbic regions involved in reward processing (ventral striatum and medial orbitofrontal cortex), whereas choices of later larger rewards were associated with relatively larger activity in fronto-parietal control regions compared to limbic regions (McClure et al., 2004, 2007; see also Turner et al., 2019). While these results were consistent with the idea of a competition between an “impulsive” and a “reflective” system, subsequent findings suggested that behavioral choices do instead rely on a common neural value signal encoded in the vmPFC (Kable and Glimcher, 2007, 2010; Hare et al., 2009, 2011; Krönke et al., 2020). While it is still debated whether self-controlled choices reflect the direct inhibition of an impulsive valuation system by the “cold” control network or whether self-control rests on the modulation of a common neural value signal (for an in-depths discussion of this debate see Goschke and Job, 2022), for our present study the important point is that both approaches assume that self-control involves top-down influences of cognitive goal representations on “hot” affective impulses.

Although such top-down influence seems highly plausible, this purely cognitive view of self-control has been challenged by theoretical and empirical studies suggesting that human decisions cannot be explained by rational and cognitive processes alone (see Phelps et al., 2014; Lerner et al., 2015 for a review). Thus, an alternative hypothesis, supported by theories of affective forecasting (e.g., Loewenstein et al., 2001; Mellers and McGraw, 2001; Gilbert and Wilson, 2007), suggests that decision making and self-control are crucially influenced by emotions directed toward long-term consequences. This implies that self-control conflicts are not only fought between reason and emotions, but rather are subject to a struggle of different emotions associated with short-and long-term goals (Kruschwitz et al., 2018a). In this sense, volitional future thinking may elicit affective anticipations of long-term consequences that could support weighing short-term versus long-term options (Pezzulo and Rigoli, 2011). For example, thoughts about the long-term costs of unhealthy eating (“I will gain weight”) may evoke negative emotions, whereas thinking about the benefits of not eating unhealthy foods (“I will stay healthy”) may evoke positive emotions. Consistent with these assumptions, we could previously demonstrate that affect-associated brain regions were simultaneously activated alongside regions of the cognitive control system when future thinking strategies were used to reduce craving for tasty but unhealthy junk food (Kruschwitz et al., 2018a). These findings may suggest that anticipated emotions are indeed incorporated into self-control-relevant deliberations with respect to possible future consequences and not only inhibited top-down by “cold cognitive processes” as implied by the “dual system” view of self-control.

In this study, we set out to gather more evidence for the impact of anticipated emotions in self-control situations. More specifically, we hypothesized that levels of self-control would be critically associated with the individual’s anticipatory ability to engage emotions associated with upcoming events. This hypothesis is fueled by the assumption that anticipated emotions may directly influence goal-directed behavior (Mellers and McGraw, 2001; Perugini and Bagozzi, 2001; Baumgartner et al., 2008; Patrick et al., 2009; Pezzulo and Rigoli, 2011). To this end, we set up a behavioral within-subject design with two independent experimental paradigms: a voluntary emotion regulation task for which we had shown in a previously fMRI task that it actually engages neural regions related to anticipating emotions (Kruschwitz et al., 2018b) and a self-control task in which subjects are confronted with a variety of everyday conflict situations measuring their ability to act self-controlled across resist temptation and endure aversion conflicts.

## 2 Methods

### 2.1 Participants

Thirty-five healthy individuals from the general population without self-reported mental disorder (recruited via email lists and a study database) participated in the experiment. Two subjects had to be excluded due to logging errors in the employed experiments, leaving the final sample with 33 subjects (19 women and 14 men, mean age = 25 years, range 18–33 years). Participants provided their written informed consent and received monetary compensation for their participation: 27 subjects received 30 Euros and 5 subjects received 35 Euros (due to recruitment difficulties during the COVID-19 pandemic monetary compensation was increased). The experiment was approved by the Ethics Committee of Technische Universität Dresden (IRB00001473).

### 2.2 General procedure

After consenting to the study, subjects participated in a within-subject design containing two experimental sessions (experiment 1: voluntary emotion regulation task; experiment 2: self-control task), which were counterbalanced in their order of completion across participants. After the two experiments, subjects were asked to complete self-report questionnaires (see below). Before leaving, subjects were debriefed and received monetary compensation for their participation.

### 2.3 Self-report questionnaires

All participants completed the Emotion Regulation Questionnaire (Abler and Kessler, 2009) that consists of two subscales measuring emotion reappraisal and emotion suppression capacities. Moreover, the brief self control scale (BSCS, Tangney et al., 2004) was completed.

### 2.4 Experiment 1 – voluntary emotion regulation task

#### 2.4.1 Experimental procedure

**Figure 1:**
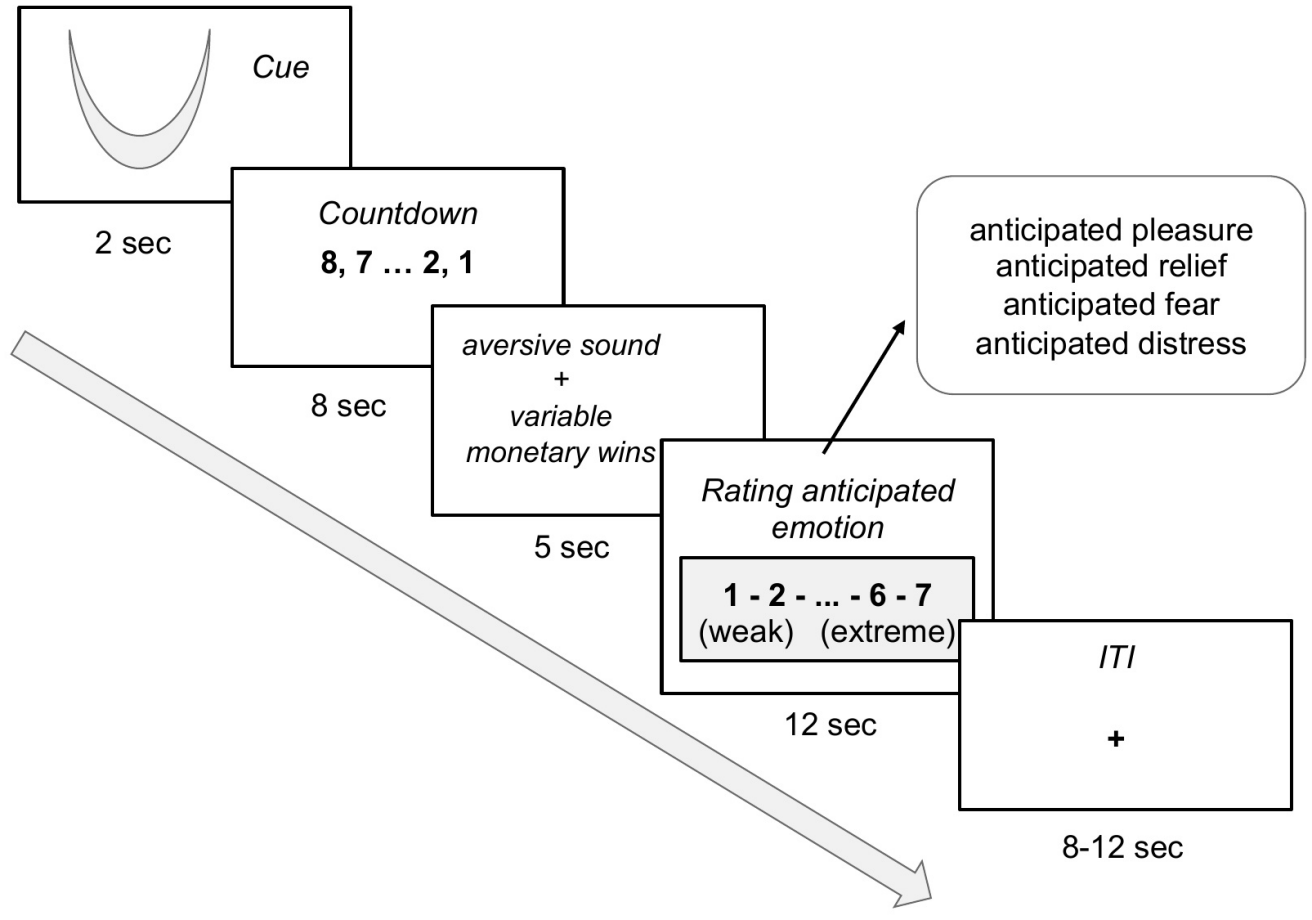
Trial sequence for experiment 1 with cues indicating to focus on the negative aspect (sound) of the bivalent outcome, to focus only on the positive aspect (money), or to focus on both aspects of the bivalent outcome.

Participants completed one run of a task with three cognitive emotion regulation strategies that we previously introduced to investigate the neural correlates of bivalent emotion anticipation (Kruschwitz et al., 2018b). In this task, participants anticipate an upcoming bivalent event consisting of 5 s of aversive sound (one of six different “natural” environmental sounds, e.g., baby crying, scratching nails on blackboard, scratching knife on plates) coupled with a monetary reward ranging from 1 cent up to 2.5 euro (on average a total of 5 euro was received and paid in addition to the fixed compensation at the end of the experiment). Emotion regulation strategies consisted of attentional deployment and were as follows: first, focus on the negative aspects (sound) of the bivalent outcome; second, focus only on the positive aspect (money); or third, focus on both aspects simultaneously. We encoded each strategy by two distinct visual cues. Prior to the experiment, memorization of the cues was verified with a quiz, which was repeated until the participant classified each cue correctly in four consecutive trials. Participants then performed a series of training trials of the task with aversive sounds that were not used in the experiment coupled to randomly assigned monetary rewards. In each trial, we instructed participants to apply one of the above mentioned voluntary emotion regulation strategies using these abstract visual cues (2 s). The instruction cue was followed by a countdown (anticipation phase; 8 s). The experimental run consisted of 36 trials (12 trials per condition) that were presented in a pseudorandomized order. In 18 of the 36 trials (pseudorandomized order) participants rated the level of anticipated emotions that they experienced during the anticipation phase (fear, distress, pleasure, relief) following the outcome on a seven point Likert scale. The rating period consisted of a total of 12 s (4 ratings with 3 s each). The inter-trial interval was varied between 8 and 12 s (Figure 1). The task was implemented with Eprime2.

#### 2.4.2 Behavioral measures of interest and task main-effects

First, to examine if participants were able to shift their anticipated emotions depending on the emotion regulation strategy (i.e., task condition), we carried out analyses for the assessment of overall task effects. Specifically, we calculated the mean of the ratings across the experiment and applied a repeated measure ANOVA for each emotion. We determined the direction of the effects via post-hoc contrasts. This analysis allowed us for example to infer whether people who focus on the negative aspects of an upcoming event are more likely to experience more anticipated fear, while people who focus on the positive aspects are more likely to experience elevated anticipated pleasure.

Second, to obtain a measure of anticipated emotion regulation capacity, we computed the difference of respective emotion ratings between the positive and the negative condition (positive > negative) for anticipated positive emotions (pleasure, relief) and vice versa for anticipated negative emotions (negative > positive; fear, distress) mimicking the approach of our previous study (Kruschwitz et al., 2018b). To derive an indicator for the general capacity to regulate anticipated emotions, we aggregated all emotion specific ratings to compute an overall anticipation score encompassing both positive and negative valence. This total score was our *a-priori* variable of interest, whereas the emotion specific ratings were used for post-hoc exploratory analyses.

Third, to further assess construct validity of the emotion anticipation task, we computed partial correlation analyses between levels of anticipated emotion regulation capacity of each emotion with the two subscales of the ERQ (covariates: age, sex, and the amount of monetary compensation).

### 2.5 Experiment 2 – self-control task

#### 2.5.1 Experimental procedure

The self-control task consisted of two parts: in a first step, here referred to as the decision part, participants were confronted with actions containing potential self-control conflicts, followed by a second rating part during which the same items had to be judged regarding their immediate and later consequences.

In the decision part, participants were instructed to indicate whether they would potentially perform a certain action in a given context. Each decision item was preceded by a context (e.g., “You are thirsty”) that remained on the screen for 3,500 ms before a potential action (e.g., “drink water”) appeared for 3,000 ms or until a response was given. Possible responses were displayed under the action statement and ranged from strong rejection to absolute agreement (strong no – no – yes – strong yes) to be indicated with one of four buttons corresponding to the spatial layout displayed on the screen. The task was implemented with Presentation.

**Figure 2:**
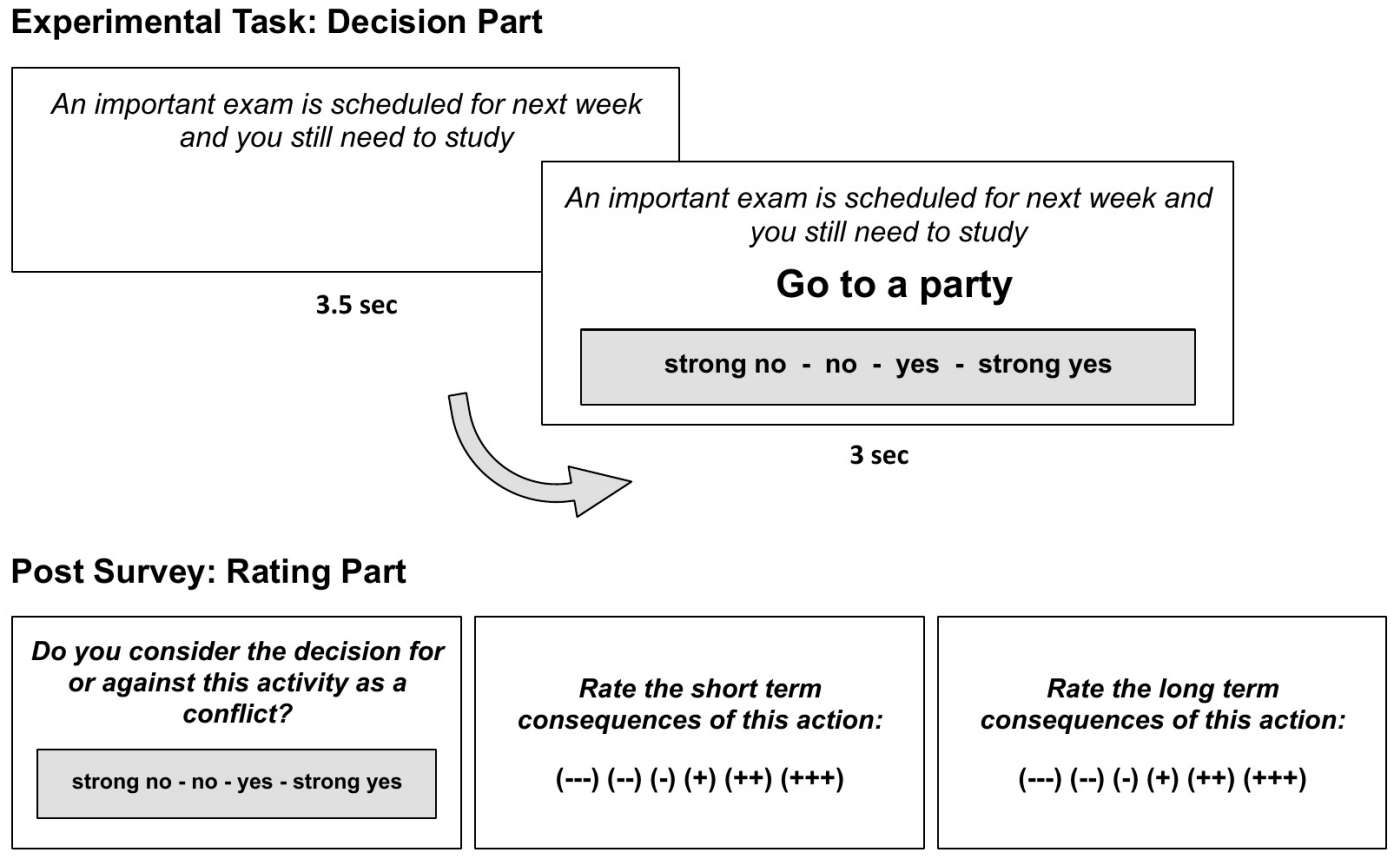
Procedure of the self-control task for an exemplary item. In the decision part (upper part of the figure) a context (“*An important exam …*”) was presented for 3.5 s. Then, a potential action (“*Go to a party*”) alongside possible responses (“*strong no – no – yes – strong yes*”) appeared for 3 s or until a response was given. After the participants answered all items in the decision part, the same items were presented again in a postsurvey for the rating part (lower part of the figure). This time, participants had to subjectively rate the extent to which they perceived this action as a conflict as well as its short and long term consequences.

After completing a total of 214 decision items in a random order, participants were asked to rate the same items in a postsurvey regarding their valence of immediate and later consequences on a six-point Likert scale ranging from very negative to very positive (−−− −− − + ++ +++). The procedure of the self-control task is depicted in Figure 2.

**Figure 3:**
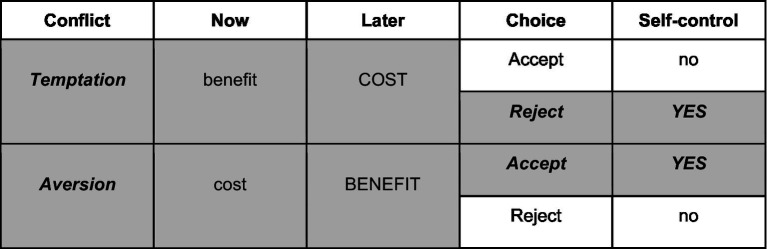
Categorization of self-control conflicts into resist temptation and endure aversion conflicts.

Based on the post-hoc rating, all decisions with immediate and later consequences ratings of opposing valence (e.g., immediate positive, later negative and vice versa) were categorized as conflicts whereas items with ratings of the same valence, i.e., immediate and later consequences both positive or both negative, were categorized as non-conflicts. Crucially, we defined items with immediate positive rating, but later negative consequences as so-called resist temptation items, whereas decision trials that were rated as immediately negative, but with later positive consequences were defined as so-called endure aversion conflicts (Figure 3).

#### 2.5.2 Behavioral measures of interest and task main-effects

First, to obtain a measure of self-control success for both types of conflict situations, respectively, (i.e., endure aversion and resist temptation conflicts), we computed self-control scores as the ratio of conflict situations with successfully applied self-control to the overall amount of conflict situations (successful self-control + self-control failures). The resulting score ranges from 0 (no self-control) to 1 (self-controlled in all conflict situations).

Second, to determine task main effects with the aim to probe whether levels of self-control as well as subjectively perceived conflict differed between endure aversion and resist temptation conflicts, we calculated paired-sample *t*-tests between both types of conflict situations.

### 2.6 Association of anticipated emotions and self-control

In order to examine if the degree to which an individual is able to regulate anticipated emotions to an upcoming event (experiment 1: voluntary emotion regulation task) would be associated to levels of self-control (experiment 2: self-control task), we performed a global linear regression with the entire data set for each self-control type (i.e., endure aversion and resist temptation). In these analyses we, respectively, employed the behavioral measures from the voluntary emotion regulation task (1 overall score, 4 separate emotion scores) as predictors of interest and included age, sex, and the amount of monetary compensation as covariates in the models. We applied Bonferroni correction for the 8 comparisons of the post-hoc emotion specific regression derived beta estimates. Furthermore, to examine if self-control can be predicted on an individual level from the level of anticipated emotions, we performed linear regressions with Leave-One-Out Cross-Validation (LOO-CV) using the same predictors and covariates as in the global regression model. Next to the general capacity to regulate anticipated emotions (anticipation total score), these analyses were conducted only for the specific associations of anticipated emotions and self-control that were significant after Bonferroni correction in the global regression model. Third, to test for associations of trait self-control to anticipatory emotion regulation capacities, we globally regressed BSCS scores with the measures from the emotion task.

#### 2.6.1 Linear regression with Leave-One-Out Cross-Validation (LOO-CV)

In our LOO-CV approach, out of the 33 data points in our sample, the model was fitted on 32 data points (training set) and applied on the one left-out data point (test case). This iterative procedure was repeated 33 times, ensuring that each data point served as a test case exactly once. For each of these iterations, a linear regression model was fit to the training set, with the emotion anticipation score as the primary predictor of interest, alongside covariates such as age, sex, and monetary status. This design allowed us to isolate the unique contribution of the emotion anticipation score in predicting self-control measures. Once the model was fitted, the standardized beta coefficient for the emotion anticipation score was derived and used to predict the self-control measure (either resist temptation or endure aversion) of the left-out test data point. Finally, we examined the correlation between the actual self-control measures and their predicted values obtained from the LOO-CV. Here, Spearman’s rank correlation was used to minimize the effect of outliers on the strength and direction of the relationship between the true and predicted scores. In its simplicity (as compared to elaborated machine-learning predictions), this procedure emphasized the robustness of our global linear model rather than claiming to provide a perfect out-of-sample generalizable individual-level prediction of the emotion anticipation scores. As in the global regression analyses, we applied Bonferroni correction for the number comparisons of the post-hoc emotion specific correlations (i.e., 4 comparisons).

#### 2.6.2 Exploratory confound analyses

To rule out the possibility that the ability to regulate anticipated emotions would align with the perception of what is considered as a conflict in the self-control task, we performed partial correlation analyses (covariates: sex, age, amount of monetary compensation) between the ability to focus on positive and negative aspects in experiment 1 with the subjective measure of perceived conflict in experiment 2. For this analysis we used the “perceived conflict rating” across conflict type (i.e., RT and EA conflicts) and further analyzed the nested structure by separating conflict ratings for situations with successful self-control versus situations with self-control failure for both domains. As such, for each conflict type there were 3 “perceived conflict ratings” (i.e., total, self-control, failure) resulting in a total of 15 correlational analyses (with anticipation total score, anticipated pleasure, relief, distress and fear as dependent variables).

## 3 Results

### 3.1 Overall task effects

#### 3.1.1 Experiment 1 – voluntary emotion regulation task

**Figure 4:**
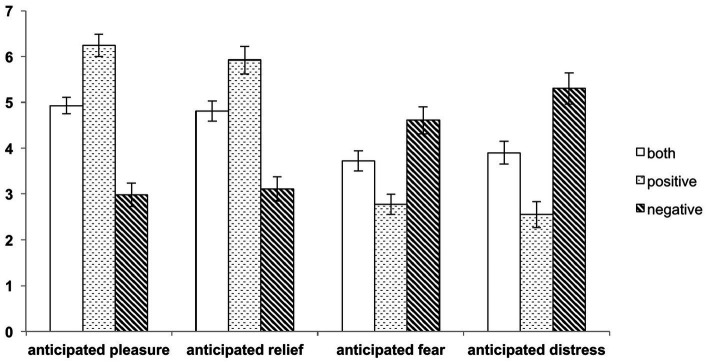
Main-effects of the emotion regulation task (experiment 1). Self-report ratings of anticipated positive and negative emotions as compared to the “anticipate both” condition show that participants were able to shift their anticipated emotions via attentional focusing depending on the respective task condition (anticipated positive: pleasure, relief; anticipated negative: fear, distress; all *p* < 0.001, error bars: standard error of the mean).

As shown in Figure 4, participants were able to shift their anticipated emotions via attentional focusing depending on the respective emotion regulation strategy (i.e., task condition). In the “anticipate positive” condition, individuals significantly experienced more positive anticipated emotions (pleasure and relief) compared to the “anticipate both” condition, where both stimuli had to be anticipated equally (see Table 1 for detailed statistics). Similarly, in the “anticipate negative” condition, there was a significant upregulation of negative emotions (fear and distress) compared to the “anticipate both” condition. Specifically, for anticipated pleasure, there was an increase of 1.31 in the “anticipate positive” condition and a decrease of 1.94 in the “anticipate negative” condition compared to the “anticipate both” condition. Regarding relief, there was an increase of 1.11 in the “anticipate positive” condition and a decrease of 1.69 in the “anticipate negative” condition. In terms of fear, there was an increase of 0.88 in the “anticipate negative” condition and a decrease of 0.94 in the “anticipate positive” condition. Lastly, for distress, the increase was 1.39 in the “anticipate negative” condition and a decrease of 1.35 in the “anticipate positive” condition. Importantly, these findings are consistent with patterns observed in our previous study (Kruschwitz et al., 2018b).

**Table 1:** Results of the repeated measures ANOVA, delineating the effect of attentional focus on the experienced anticipated emotion in experiment 1.

Emotion	*F*-value	*p*-value	*η* ^2^	BOTH condition	POSITIVE condition	NEGATIVE condition
Pleasure	55.69	< 0.001	0.635	4.93 (± 0.18)	6.24 (± 0.24)	2.98 (± 0.25)
Relief	41.29	< 0.001	0.563	4.81 (± 0.22)	5.92 (± 0.3)	3.11 (± 0.26)
Fear	14.69	< 0.001	0.315	3.72 (± 0.22)	2.77 (± 0.22)	4.61 (± 0.3)
Distress	30.95	< 0.001	0.492	3.9 (± 0.25)	2.55 (± 0.28)	5.3 (± 0.34)

For each condition (“both,” “positive,” “negative”) the mean rating (± standard error) of the experienced anticipated emotion is shown. All post-hoc comparisons for the emotion specific comparisons of “positive>both” and “negative>both” were statistically significant (all *p* < 0.001).

Moreover, the ability to regulate anticipated emotions for an upcoming event was found to be associated with the ERQ subscale “reappraisal”. For positive emotions, the direction of the applied contrast was positive > negative, and vice versa for negative emotions (pleasure: *r* = 0.447, *p* = 0.013; relief: *r* = 0.356, *p* = 0.053; fear: *r* = 0.478, *p* = 0.008; distress: *r* = 0.384, *p* = 0.036). No significant associations were found between the capacity of regulating anticipated emotions and the ERQ subscale “supression” (pleasure: *r* = −0.100, *p* = 0.599; relief: *r* = −0.017, *p* = 0.931; fear: *r* = −0.270, *p* = 0.150; distress: *r* = −0.123, *p* = 0.518).

#### 3.1.2 Experiment 2 – self control task

**Figure 5:**
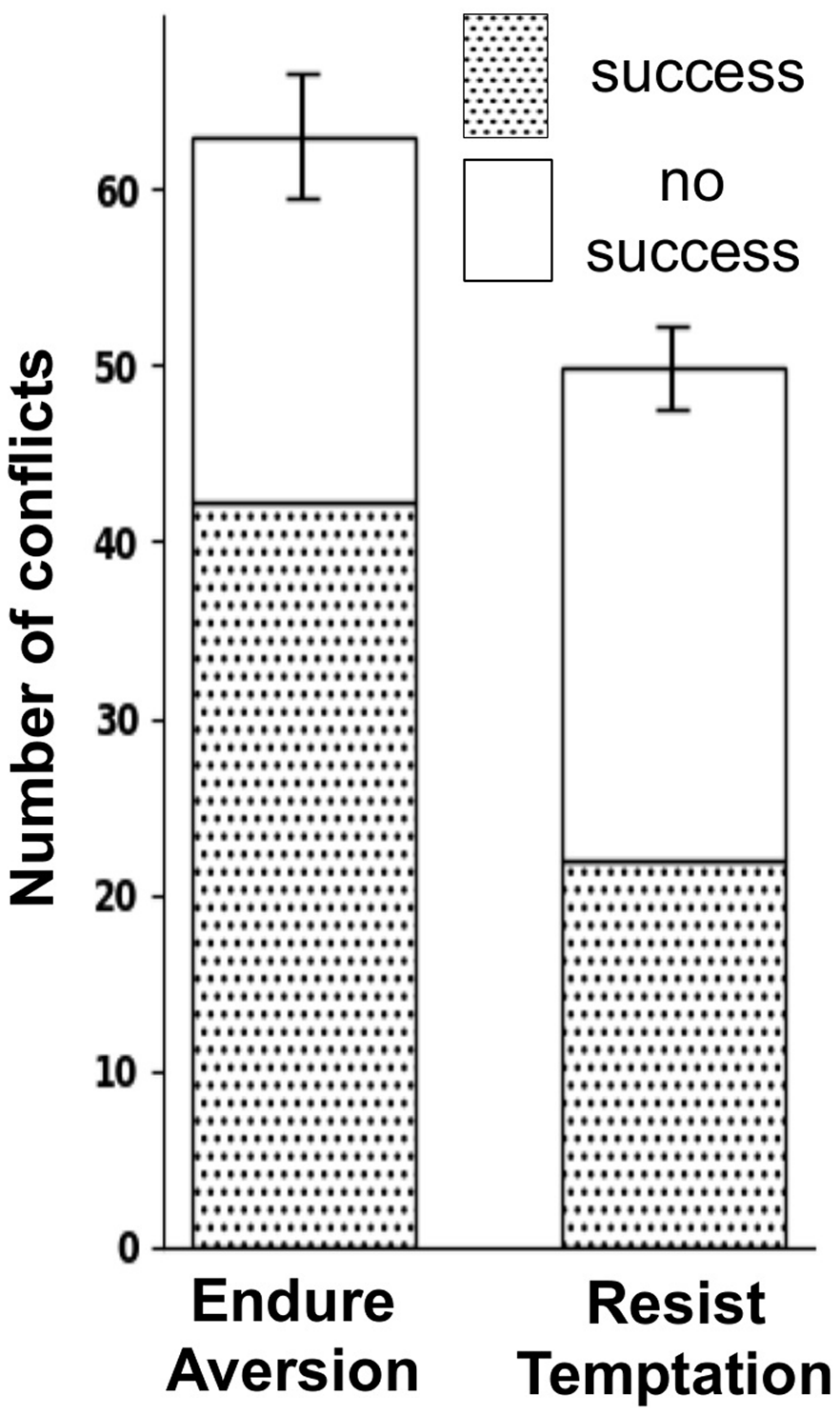
Number of self-control conflicts and successfully applied self-control according to the type of conflict. Specifically, participants experienced more endure aversion (EA) conflicts than resist temptation (RT) conflicts [number of EA conflicts: 63.0 (± 3.5), number of RT conflicts: 49.9 (± 2.3)] and also demonstrated a greater ability to apply self-control during endure aversion conflicts with a success rate of 0.67 (± 0.021), compared to a success rate of 0.44 (± 0.022) during resist temptation conflicts.

As shown in Figure 5, participants encountered a higher number of endure aversion conflicts compared to resist temptation conflicts, as reflected in the mean values of 63.0 and 49.9, respectively. Participants also demonstrated a greater ability to apply self-control during endure aversion conflicts with a success rate of 0.67, compared to a success rate of 0.44 during resist temptation conflicts. Both differences were statistically significant (*p* < 0.001). In line with this trend, individuals subjectively perceived conflicts as higher in resist temptation (RT) as compared to endure aversion (EA) conflicts (RT: 2.619; EA: 2.152; *p* = 0.003). Within endure aversion conflicts the perceived conflict strength was higher in situations of self-control failure as in situations where self-control was applied successfully (success: 2.019, failure: 2.458; *p* = 0.001). Within resist temptation conflicts the conflict strength was slightly higher in situations where self-control was applied successfully as compared to situations with self-control failure (success: 2.666, failure: 2.600; *p* = 0.01).

### 3.2 Association of anticipated emotions and self-control

In order to examine if the degree to which an individual is able to regulate anticipated emotions to an upcoming event (experiment 1: voluntary emotion regulation task) is associated to levels of self-control (experiment 2: self-control task), we performed a global linear regression with the entire data set for each self-control type (i.e., endure aversion and resist temptation). The same type of analysis was conducted with scores of the BSCS questionnaire to probe for associations of anticipated emotions and trait self-control. Second, to examine if self-control can be predicted on an individual level from the level of anticipated emotions, a linear regression with Leave-One-Out Cross-Validation (LOO-CV) was conducted on the significant emotion/self-control associations from the global regression and correlations of true versus predicted self-control scores were computed.

#### 3.2.1 Global linear regression of anticipated emotions and self-control

**Figure 6:**
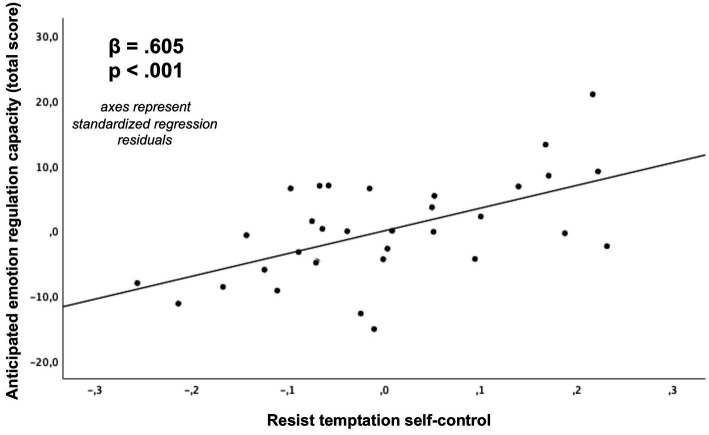
Association of self-control in resist temptation conflicts with the general capacity to regulate anticipated emotions (total score, *p*-value not Bonferroni corrected due to *a-priori* hypothesis) derived from a multiple regression analysis (covariates: age, sex, monetary compensation).

**Figure 7:**
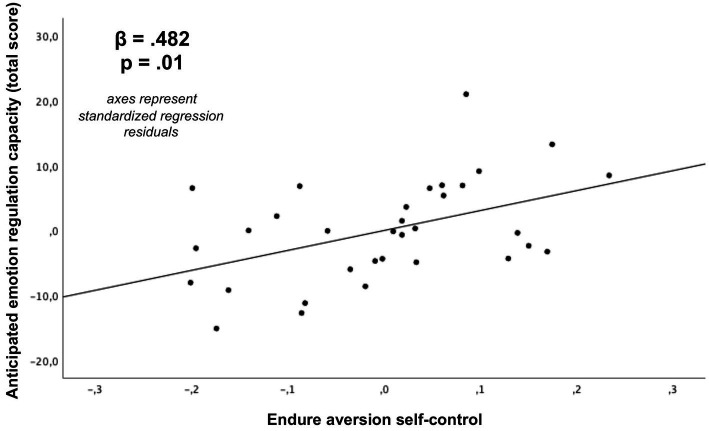
Association of self-control in endure aversion conflicts with the general capacity to regulate anticipated emotions (total score, *p*-value not Bonferroni corrected due to *a-priori* hypothesis) derived from a multiple regression analysis (covariates: age, sex, monetary compensation).

**Figure 8:**
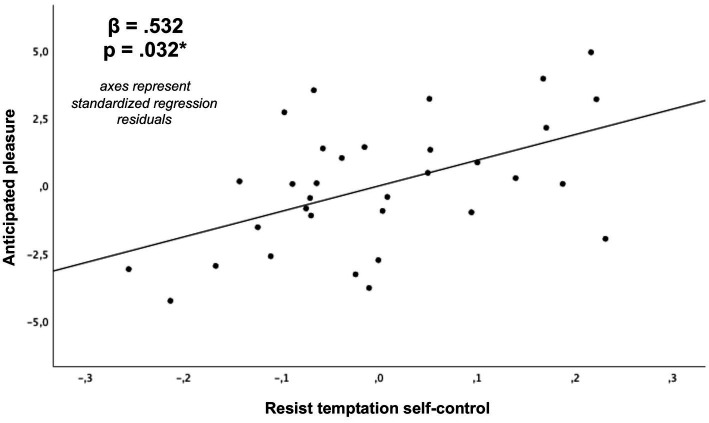
Association of self-control in resist temptation conflicts with the capacity to regulate anticipated pleasure (*p-value Bonferroni corrected for 8 post-hoc comparisons) derived from a multiple regression analysis (covariates: age, sex, monetary compensation).

**Figure 9:**
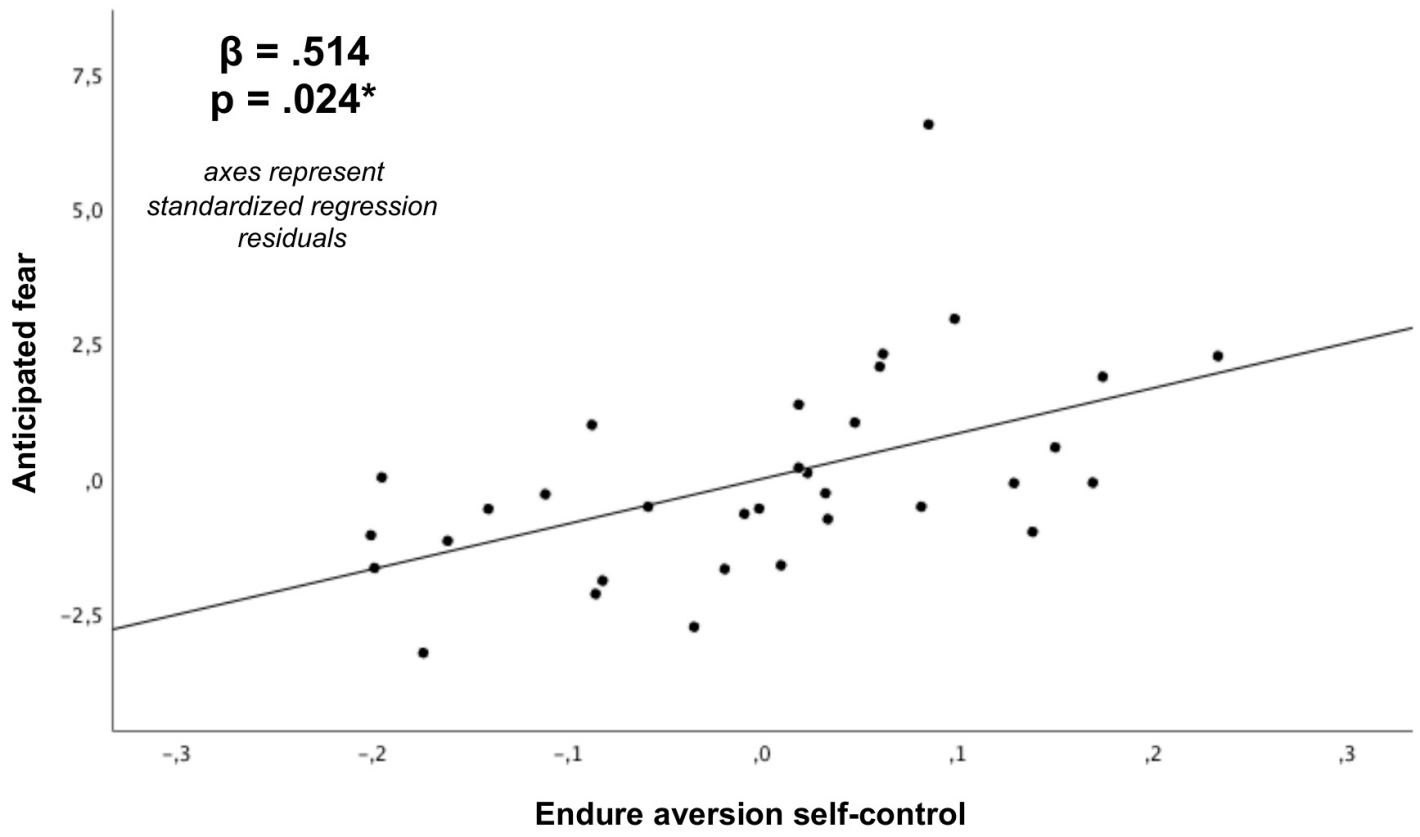
Association of self-control in endure aversion conflicts with the capacity to regulate anticipated fear (*p-value Bonferroni corrected for 8 post-hoc comparisons) derived from a multiple regression analysis (covariates: age, sex, monetary compensation).

As depicted in Table 2 and Figures 6–9, global linear regression analyses (controlling for the covariates of age, sex and monetary compensation) revealed significant associations for both types of self-control with the general capacity to regulate anticipated emotions (resist temptation with 𝛽 = 0.605, *p* < 0.001; endure aversion with 𝛽 = 0.482, *p* = 0.01). With respect to emotion specific regulation capacities, we post-hoc found significant Bonferroni corrected associations for self-control in resist temptation conflicts with regulation capacities of anticipated pleasure (𝛽 = 0.532, *p*-corrected = 0.032; *p*-uncorrected = 0.004; Figure 8), anticipated fear (𝛽 = 0.576, *p*-corrected = 0.008, *p*-uncorrected = 0.001), and anticipated distress (𝛽 = 0.607, *p*-corrected = 0.008, *p*-uncorrected = 0.001). The association between self-control in resist temptation conflicts and regulation of anticipated relief did not remain significant after Bonferroni correction (𝛽 = 0.464, *p*-corrected =0.152, *p*-uncorrected =0.019). For self-control in endure aversion conflicts, we only observed a significant Bonferroni corrected association with regulation of anticipated fear (𝛽 = 0.514, *p*-corrected = 0.024, *p*-uncorrected = 0.003; Figure 9), whereas associations to anticipated pleasure (𝛽 = 0.466, *p*-corrected = 0.96, *p*-uncorrected = 0.012), and anticipated distress (𝛽 = 0.413, *p*-corrected = 0.24, *p*-uncorrected = 0.030) did not remain significant after multiple comparisons correction. Anticipated relief was not associated significantly to self-control in this conflict type (𝛽 = 0.340, *p*-uncorrected = 0.088). We did not observe any significant associations between scores of the BSCS and task specific emotion anticipation ratings (all *p* > 0.05).

**Table 2:** Results of the 10 global linear regression analyses between both tasks (predictor of interest: level of anticipated emotion; dependent variable: self-control in resist temptation or endure aversion conflicts respectively; covariates: age, gender, monetary compensation).

Anticipation	Resist temptation self-control		Endure aversion self-control	
	𝛽	*p*-value	𝛽	*p*-value
General emotion anticipation (total score)	0.605	< 0.001	0.482	0.01
Pleasure	0.532	0.004, 0.032*	0.466	0.012, 0.96*
Relief	0.464	0.019, 0.152*	0.340	0.088, 1*
Fear	0.576	0.001, 0.008*	0.514	0.003, 0.024*
Distress	0.607	0.001, 0.008*	0.413	0.030, 0.24*

Significant associations for both types of self-control with the general capacity to regulate anticipated emotions were found. Associations with emotion specific regulation capabilities were analyzed post-hoc, both uncorrected and Bonferroni-corrected* *p*-values are shown.

#### 3.2.2 Analysis with LOO-CV for individual prediction of self-control from anticipated emotions

**Figure 10:**
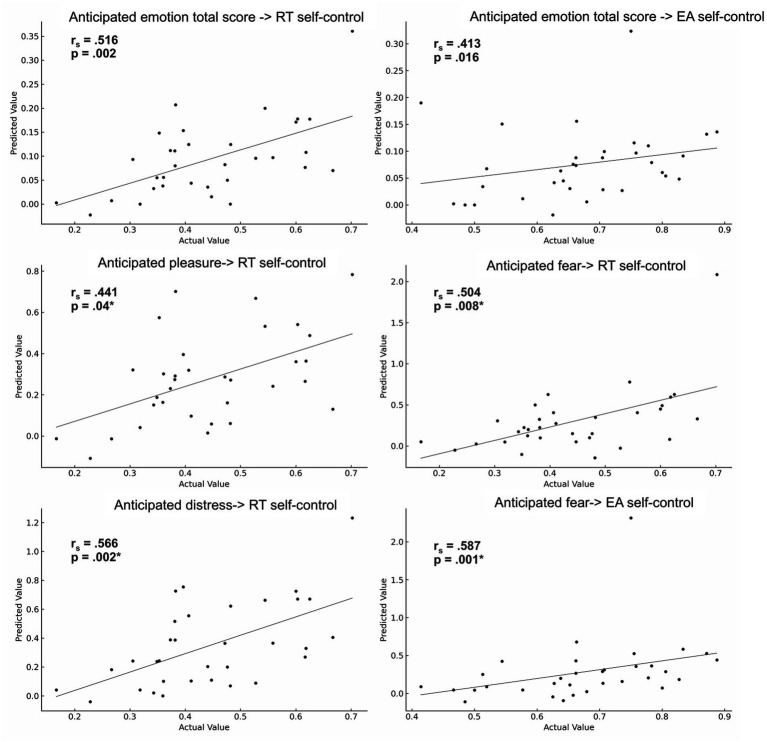
Association of true and predicted self-control scores for resist temptation (RT) and endure aversion conflicts (EA) as a result from the Leave-One-Out Cross-Validation (LOO-CV) multiple linear regression approach (*p-value Bonferroni corrected for 4 comparisons).

Based on the results from the global regression analyses, we conducted the LOO-CV prediction approach of individual resist temptation self-control scores with the general capacity to regulate anticipated emotions (total score), anticipated pleasure, anticipated fear and anticipated distress. Prediction of self-control scores in endure aversion conflicts were conducted with the anticipation total score and anticipated fear. Based on the results from the global regression analyses, we conducted the LOO-CV prediction approach of individual resist temptation self-control scores with the general capacity to regulate anticipated emotions (total score), anticipated pleasure, anticipated fear and anticipated distress. Prediction of self-control scores in endure aversion conflicts were conducted with the anticipation total score and anticipated fear. As shown in Table 3, these analyses revealed significant (uncorrected) correlations between true and predicted self-control scores for the anticipation total score in both conflict types (all *p* < 0.05), as well as significant Bonferroni corrected predictions of self-control scores for all exploratory emotion specific measures. Specifically, in resist temptation conflicts effects were observed for anticipated distress (*r*_s_ = 0.56, *p*-corrected = 0.002), anticipated fear (*r*_s_ = 0.50, *p*-corrected = 0.008) and anticipated pleasure (*r*_s_ = 0.44, *p*-corrected = 0.04). For the prediction of self-control in endure aversion conflicts, we observed a significant association with the regulation of anticipated fear (*r*_s_ = 0.58, *p*-corrected = 0.001). Notably, the narrow range in predictions for some emotion measures is a reflection of the predictor’s partial coverage of the outcome’s variance and may also be due to the simplicity of the linear model (as compared to more elaborated prediction approaches with hyperparameter-tuning). In conclusion, our methodological approach emphasizes on the robustness of the global linear regression and pinpoints the strength and direction of the relationship between true and predicted values on an individual level. Figure 10 illustrates the relationship of true and predicted self-control scores for all LOO-CV predictions.

**Table 3:** Association of true and predicted self-control scores for resist temptation (RT) and endure aversion conflicts (EA) as a result from the Leave-One-Out Cross-Validation (LOO-CV) multiple linear regression approach.

Prediction of self-control from anticipated emotion	Spearman correlation (*r*_s_)	*p*-value
Anticipation emotion total score → RT self-control	0.51	0.002
Anticipation emotion total score → EA self-control	0.41	0.016
Pleasure → RT self-control	0.44	0.01, 04*
Fear → RT self-control	0.50	0.002, 0.008*
Fear → EA self-control	0.58	< 0.001, 0.001*
Distress → RT self-control	0.56	< 0.001, 0.002*

Both uncorrected and Bonferroni-corrected* *p*-values are shown (4 comparisons of post-hoc emotions across RT and EA conflicts).

#### 3.2.3 Exploratory confound analyses

To test an alternative explanation for our proposed association of anticipated emotions and self-control, namely that the ability to regulate anticipated emotions would align with the perception of what is considered as a conflict in the self-control task, we performed partial correlation analyses (covariates: sex, age, amount of monetary compensation) between the ability to focus on positive and negative aspects in experiment 1 with the subjective measure of perceived conflict in experiment 2. Among these analyses we only observed an uncorrected significant negative association of anticipated distress with the perceived conflict strength in endure aversion conflicts in which self-control was applied successfully (*r* = −0.416, *p* = 0.02). All other results had below threshold (uncorrected) significance levels (all *p* > 0.05). The only observed association was however extremely insignificant after correcting for multiple-testing. Given these results we would assume that the ability to shift focus in the emotion regulation task does not align with the perception of what is considered as a conflict during self-control.

## 4 Discussion

Following the notion that anticipated emotions may directly influence goal-directed behavior, we set out to test if self-control would be affected by the individual’s anticipatory ability to mobilize emotions associated with upcoming events. To this end, we used a within-subject design with two independent experimental paradigms: a voluntary emotion regulation task in which participants were instructed to control their anticipated emotions by selectively attending either to the positive or negative aspects of an anticipated bivalent event and a self-control task in which subjects were confronted with a variety of everyday conflict situations measuring their ability to act self-controlled in resist temptation and endure aversion conflicts. By regressing behavioral measures across these experiments we found that (i) individuals who were better able to generally engage anticipated emotions to an upcoming event showed stronger levels of self-control in all conflict situations. (ii) Individuals who were better able to engage positive and negative anticipated emotions to an upcoming event showed stronger levels of self-control in situations where they had to resist temptations in order to achieve a long-term goal. (iii) For situations requiring to endure aversive short-term consequences to achieve long-term goals we observed associations between the ability to engage negative anticipated emotions and levels of self-control. These findings suggest that self-control is directly linked to the capacity of engaging emotions associated with future events.

The ability to associate actions with their consequences is a crucial prerequisite for self-control, as it enables individuals to pursue goals that are not only motivated by current needs and impulses but rather anticipated future needs (Kuhl and Goschke, 1994). Specifically, long-term goals are often in conflict with impulsive reactions or current needs and self-control is required to resist these immediate temptations, inhibit impulsive reactions, and accept short-term costs (Hofmann et al., 2009a; Mischel et al., 2011). A standard example is, when the intention to follow a diet is undermined by the sight of a tasty dessert. In this context, the predominant view on self-control would assume that self-control rests on “cold” cognitive goal representations associated with the long-term outcomes of the diet (e.g., staying healthy and not gaining weight) that influence or compete top-down with affective impulses (e.g., the tasty desert) arising in “hot” affective brain regions (McClure et al., 2007; Hare et al., 2009, 2011). This view on self-control is however challenged by the notion that human decisions often cannot be explained by rational and cognitive processes alone but are considerably influenced by emotions directed towards long-term consequences (Loewenstein et al., 2001; Mellers and McGraw, 2001; Gilbert and Wilson, 2007; Phelps et al., 2014; Lerner et al., 2015). That is, thoughts about the long-term costs of unhealthy eating (“I will gain weight”) may evoke negative emotions, whereas thinking about the benefits of not eating unhealthy foods (“I will stay healthy”) may evoke positive emotions. These affective anticipations of long-term consequences could in turn support weighing short-term versus long-term options (Pezzulo and Rigoli, 2011) and imply that self-control conflicts are not only fought between reason and emotions, but are also subject to a struggle of different emotions associated with short-and long-term goals (Kruschwitz et al., 2018a). Consistent with these assumptions, we observed that individuals who were generally better able to engage anticipated emotions with respect to an upcoming event showed stronger levels of self-control in situations where they had to resist temptations or to endure aversions in order to achieve a long-term goal. In post-hoc analyses, we observed that the ability to engage both, positive and negative anticipated emotions was beneficial for self-control in situations where temptations had to be resisted (e.g., resisting a tasty but unhealthy desert), whereas the engagement of anticipated negative emotions led to more self-control in situations where it was necessary to endure a short term aversive state (e.g., getting up to exercise after a hard day at work). Most importantly, to further elucidate the practical implications of these findings, we employed a Leave-One-Out Cross-Validation (LOO-CV) prediction approach alongside our global linear regression analyses. While the global regression identified significant associations between anticipated emotions and self-control measures, the LOO-CV approach was instrumental in assessing the predictive power of these associations at an individual level. This dual-methodology framework not only confirmed the robustness of our findings but also highlighted their potential applicability in personalized predictive models. Such an approach is essential in psychological research where the ultimate goal often extends beyond understanding general trends to include reliable individual-level predictions and interventions.

Although this is the first study demonstrating a direct association of self-controlled behavior and the ability to engage anticipated emotions with upcoming events, links between affective forecasting and self-control have been shown already by previous studies (e.g., Mellers and McGraw, 2001; Perugini and Bagozzi, 2001; Bagozzi et al., 2003; Idson et al., 2004; Hynie et al., 2006; Patrick et al., 2009; Kotabe et al., 2019). For example, anticipated negative emotions associated with goal failure were shown to correlate with intentions to achieve self-control goals (Bagozzi et al., 2003), whereas anticipated positive emotions associated with goal achievement correlated with intentions to diet and exercise (Perugini and Bagozzi, 2001). More recently, Kotabe et al. (2019) proposed that anticipated emotions are key in guiding self-control judgements and provided evidence for a relatively strong weighting of anticipated guilt and relatively weak weighting of anticipated pride in these judgements. In line with the stronger prominence of negative anticipated emotions, we observed a substantial attenuation of significant results for positive anticipated emotions and their associations with self-control after applying Bonferroni correction (in the global regression and individual prediction approach). This differential impact of anticipated emotions on self-control could suggest a potential overestimation in the initial associations of positive emotions with self-control outcomes or alternatively reflects the inherent asymmetry in the motivational forces of negative versus positive emotional states during self-control processes. The latter interpretation aligns with the concept of a negativity bias, where the motivational pull of negative emotions such as fear or distress may tend to surpass that of positive emotions like pleasure or relief in self-regulatory processes (*cf.* Tversky and Kahneman, 1991; Baumeister et al., 2001; Rozin and Royzman, 2001). Therefore, the robust association of negative anticipated emotions with self-control, even after stringent statistical adjustments, lends credence to the notion that individuals may be more attuned to the regulatory influence of potential negative outcomes. This is not to undermine the role of positive anticipations but to acknowledge that their influence may be subtler and possibly overshadowed by the negative consequences in self-control contexts. For example, we observed that the engagement of anticipated negative emotions not only led to more self-control in temptation conflicts but also in situations where it was necessary to endure a short-term aversive state. Although one may expect that anticipating positive long-term effects are of benefit to overcome a short-term aversive state, this theorized negativity bias may be the reason for the relatively stronger association of anticipated negative emotions in aversion conflicts (e.g., thinking about gaining weight when not exercising after a hard day at work). Despite these speculations, our findings invite a critical examination of the measures used to assess the regulation of anticipated emotions. The discrepancy in significance could suggest that our measures may be more sensitive to detecting the influence of negative emotions. It also prompts a consideration of alternative methodologies that might yield more nuanced insights into the complex interplay between emotional anticipation and self-control, particularly for positive emotions.

Other evidence for links between affective forecasting and self-control are also present in our own previous work. Specifically, in one of our prior experiments participants were instructed to regulate their craving by thinking of the positive consequences of resisting, or the negative consequences of not resisting tasty but unhealthy junk food (Kruschwitz et al., 2018a). In a control condition, they anticipated the pleasure of eating and thus, allowed the craving to occur. When contrasting these conditions, we could demonstrate that affect-associated brain regions were simultaneously activated alongside regions of the cognitive control system when future thinking strategies were used to exert craving related self-control. Although such co-activation does not allow drawing inferences about its mechanism for self-control, we found that activation in the exact same brain regions correlated with anticipated affect in a similar experimental setup as employed in this current study (Kruschwitz et al., 2018b). We interpreted these findings such that “hot,” affective processes may, at least in certain circumstances, play a role in self-control. In two of our more recent studies (Kruschwitz et al., 2019; Walter et al., 2020) we employed an inspiratory breathing restriction task that evokes strong negative emotions and could furthermore demonstrate that individuals who “over-estimated” their upcoming interoceptive state with respect to experienced dyspnea (i.e., anticipated versus experienced) were more effective in the down-regulation of craving using negative future-thinking strategies. In both studies, these individuals also obtained higher scores on a measure of trait self-control, i.e., self-regulation to achieve long-term goals. As some theories assume that interoceptive prediction errors can give rise to subjective feeling states (Seth and Critchley, 2013) these previous findings may indirectly point towards associations between anticipated affect and self-control. Also studies from other research groups have pointed in this direction: for instance, there is converging evidence from neuroimaging studies that farsighted decisions may be supported by the integration of episodic simulations of future outcomes with their emotional quality. At a neural level, there is evidence that self-controlled choices in tasks involving conflicts between short-and long-term outcomes rest on the top-down modulation of evidence accumulation and value integration processes in the ventromedial prefrontal cortex (vmPFC) by anticipated long-term outcomes (Kable and Glimcher, 2007, 2010; Hare et al., 2009, 2011; Krönke et al., 2020). Of direct relevance for the present study, the vmPFC has also been implicated in episodic prospection and the imaging of future events and appears to contribute to affective forecasting by integrating representations of future episodes with their anticipated affective quality (Benoit et al., 2014). Such a mechanism is consistent with our present findings and suggests that the integration of imagined future episodes with anticipatory emotions renders future outcomes vivid and salient, thereby supporting self-controlled choices.

From a more general perspective, anticipatory emotions can be considered a key element of the ability to anticipate one’s own future motivational states (Goschke, 2013) and to take the perspective of one’s future self (Urminsky, 2017). Evidence from a TMS study indicates that a brain region involved in perspective taking (the right temporo-parietal junction, TPJ) is also causally involved in making farsighted decisions, presumably by supporting the imaging of affective-somatic states of one’s future self (Soutschek et al., 2016). Of note, in our above-mentioned own previous work (Walter et al., 2020), we found that higher self-control in a craving regulation task was associated with increased connectivity in a network including regions of the cognitive control network as well as the right TPJ. Moreover, we obtained a correlation between activation in the TPJ and interoceptive predictions in a breathing restriction task. While further research is clearly required to elucidate the functional relation between anticipatory emotions, interoceptive predictions, and perspective taking, together with our present findings these results are consistent with the hypothesis that anticipatory emotions support self-control by rendering the perspective of one’s future self tangible and motivationally salient at the moment of decision. Based on these reviewed studies it appears that the engagement of anticipated emotions or affective states associated with long-term outcomes provide a common ground for various aspects of self-control.

Taken together, our findings may suggest that anticipated emotions are indeed incorporated into self-control-relevant deliberations with respect to possible future consequences and not only inhibited top-down by “cold cognitive processes” as implied by the “dual system” view of self-control. When making a choice, we need to anticipate future affective states linked to the outcomes of the different alternatives and weigh them to the short-term options. Only if we are in a position to fully engage in the affective consequences of a decision, can we effectively support self-control by modulating a shared value signal to become congruent with long-term goals (Hare et al., 2009, 2011).

This study contains limitations. First, since the experiment on the anticipation of bivalent stimuli involved monetary rewards and unpleasant sounds, observed emotion ratings can, strictly speaking, only be interpreted in terms of the anticipation of these specific stimuli and not in terms of the processing of general valence as well as its influence in self-control situations. However, this criticism is countered by our previously identified underlying neural correlates (Kruschwitz et al., 2018b) and other studies demonstrating that that neural activity in the ventral striatum, vmPFC, PCC (monetary win) and insula (aversive noise) is related to the processing of general positive and negative valence (e.g., Knutson and Greer, 2008; Benoit et al., 2011, 2014). Thus, it stands to reason that the measured anticipatory emotion regulation capacity does not specifically relate only to the stimuli used in the experiment. A second criticism of the experiment lies in the repeated presentation of the same experimental cues used to announce monetary reward and unpleasant sounds, respectively. The repeated presentation could have led to conditioning effects, which would mean that the presentation of the cue stimulus already evoked emotions that were subsequently no longer purely anticipatory in nature. As this is a general problem of studies on anticipation (e.g., Carlson et al., 2011), future studies should devise experimental designs that could circumvent this problem, for example, by using different cues with the same meaning. Third, the experimental design of the emotion regulation task was limited by theoretical restraints derived by the research question itself. Specifically, all ratings were performed after participants experienced the actual outcome, which could have influenced the rating of the anticipated emotions. However, from a theoretical point of view, anticipated emotions can be strongly biased by the subjective uncertainty on whether a specific event will occur or not (i.e., uncertainty may modulate levels of anticipated emotion; Baumgartner et al., 2008). Therefore, it was necessary for outcomes to occur directly after the anticipation period. Consequently, it remains the possibility that subjective ratings were also influenced by the actual stimulus outcome and did not uniquely represent the level of anticipation (e.g., self-report ratings of anticipated emotions could be driven by the experienced outcome). As the current task-design does not allow ruling out this influence, future work with alternative task-designs may be conducted to investigate potential outcome effects on the rating of anticipated emotions. To stay in line with our previous work (cf. Kruschwitz et al., 2018b) and for replication purposes, we did not modify the task design in this current study. Fourth, interpretations of results in our experiment are limited to the combination of aversive sound and monetary reward. Therefore, the possibility remains that the observed effects specifically depict orienting towards or away from unpleasant noise or monetary reward rather than the influence of anticipating valence in general. However, in our prior work with the exact same version of this experiment in an fMRI context, we observed activation of the ventral striatum and insula during the anticipation, which are brain regions implicated in the general processing of rewarding and negative stimuli respectively, as well as activations of ventro-medial prefrontal and posterior cingulate cortices that are generally associated with future thinking (Kruschwitz et al., 2018b; c.f. experiment 2). Fifth, we did not observe significant associations between anticipatory emotion ratings with trait self-control (BSCS). In this context it must be stated that a recent report of a Bayesian correlational analysis also revealed little-to-no relationships between self-reported self-control and performance on laboratory tasks of inhibitory control (Stroop and Flanker tasks) (Saunders et al., 2018). This lack of correlation does not invalidate these measures, but indicates that different indicators of the construct self-control do not converge. Self-report measures assess a generalized subjective judgment about how frequently one behaves in a self-controlled manner (i.e., it measures the outcome of self-control processes), but self-reports may not differentiate between different mechanisms underlying self-controlled behavior. Whereas interventive self-control strategies like craving regulation or the generation of anticipatory emotions primarily play a role when one faces a temptation and cannot avoid a self-control conflict, there is evidence that self-control in real-life contexts often depends on the formation of beneficial habits (Galla and Duckworth, 2015; Gillebaart and de Ridder, 2015; De Ridder and Gillebaart, 2017; Gillebaart and Adriaanse, 2017) or preventive precommitment strategies that serve to avoid temptations (Kurth-Nelson and Redish, 2012; Soutschek et al., 2017; Studer et al., 2019). This may explain why self-reported trait self-control and interventive strategies like anticipatory emotions are often not strongly correlated.

In summary, this study challenges the conventional “dual system” view of self-control by demonstrating that the ability to anticipate and engage with emotions towards future events plays a crucial role in self-control. Through a within-subject design involving an emotion regulation and self-control task, it was found that individuals who better engage anticipated emotions exhibit stronger self-control in resisting temptations and enduring aversions for long-term goals. These findings suggest a more complex interplay of cognitive and emotional processes in self-control than previously understood. Looking ahead, this research opens up exciting possibilities for future studies to explore how individual differences in emotional anticipation affect various aspects of decision-making and goal achievement, which may pave the way for developing new behavioral interventions and psychological therapies that harness the power of anticipated emotions.

## Data availability statement

The original contributions presented in the study are included in the article/Supplementary material, further inquiries can be directed to the corresponding author/s.

## Ethics statement

The studies involving humans were approved by Ethics Committee of Technische Universität Dresden (IRB00001473). The studies were conducted in accordance with the local legislation and institutional requirements. The participants provided their written informed consent to participate in this study.

## Author contributions

JK was involved in the conception/design of the work, analysis, and interpretation of data, and in writing the article. TG was involved in the conception/design of the work, interpretation of results and in writing the article. EA was involved in the data acquisition. A-CK was involved in the analysis, and interpretation of data, and in revising the article. FK and HW were involved in the conception/design of the work and in interpreting the results. All authors contributed to the article and approved the submitted version.
